# Highlights of the 2 ^nd^ Bioinformatics Student Symposium by ISCB RSG-UK

**DOI:** 10.12688/f1000research.8445.1

**Published:** 2016-05-17

**Authors:** Benjamen White, Vayani Fatima, Nazeefa Fatima, Sayoni Das, Farzana Rahman, Mehedi Hassan

**Affiliations:** 1 The Genome Analysis Centre, Norwich, UK; 2 Algorithms and Bioinformatics Group, King's College London, London, UK; 3 Eukaryotic Evolution Research Group, University of Huddersfield, Huddersfield, UK; 4 Research Department of Structural and Molecular Biology, University College London, London, UK; 5 School of Computing and Mathematics, University of South Wales, Cardiff, UK

**Keywords:** RSG-UK, ISCB, ISCBSC, student, symposium, bioinformatics, computational biology

## Abstract

Following the success of the 1 ^st^ Student Symposium by ISCB RSG-UK, a 2 ^nd^ Student Symposium took place on 7 ^th^ October 2015 at The Genome Analysis Centre, Norwich, UK. This short report summarizes the main highlights from the 2 ^nd^ Bioinformatics Student Symposium.

## Background

The United Kingdom Regional Student Group (RSG-UK) of the International Society for Computational Biology (ISCB) is part of a global network of students and early-stage post-doctoral researchers. The non-profit group was formed in December 2013, with a vision to build, strengthen and enhance a network among young computational biologists working and studying in the United Kingdom. The founders took inspiration from similar initiatives and examples set by the ISCB Student Council (ISCBSC) and its regional groups ^1– 3^. The group strongly believes that the student symposiums and future events will help us to improve the pipeline for the professional development of future computational biologists. RSG-UK welcomes students and early career researchers from all areas of computational biology to join our community.

Our current leadership and members include research students and post-doctoral researchers from computational biology and other life sciences. The community provides opportunities for networking, soft skills enhancement for both undergraduates and postgraduates, and developing relationships between young computational biologists and academic/industry partners within the UK.

## 2 ^nd^ Bioinformatics Student Symposium

The 2 ^nd^ Bioinformatics Student Symposium was held on 7 ^th^ October 2015 at The Genome Analysis Centre, UK. The symposium continued the format from the previous year, drawing from the workshops and conferences arranged by the ISCB Student Council and its regional student groups across the globe ^1, 3– 5^. Our event was kindly supported by: The Genome Analysis Centre, a BBSRC strategically-funded research institute; Illumina, Inc., a global developer and manufacturer of technology for genetics research; Repositive, a data platform provider for advancing genetic diagnostics and research; and *F1000Research*, an open access peer-reviewed scientific publishing platform.

### Scope and format of the meeting

The symposium aimed to be a forum for students of all levels and early-career researchers in the fields of computational biology and bioinformatics. Our aim was achieved by providing the opportunities for delegates to network and exchange knowledge with senior researchers, and also representatives from industry. The traditional scientific component of the meeting involved four sessions of oral presentations by students and post-doctoral researchers. The sessions also included three senior researchers as keynote speakers, a careers presentation and a scientific presentation from the ISCB Student Council founder, Dr. Manuel Corpas.

Prof. Dylan Edwards, Interim Director of The Genome Analysis Centre and Chair of Cancer Studies in the School of Biological Sciences at the nearby University of East Anglia, gave a welcoming address to the students and invited the chairs to begin the Symposium.

The RSG UK committee received more than 20 abstracts from students who wished to present their work at the Symposium. The submitted abstracts were peer reviewed by senior and junior researchers from different UK affiliations. The reviewers selected a total of six abstracts for oral presentations, and twelve abstracts for poster presentations. Overall, 50 delegates from 29 UK institutions attended the Symposium with international delegates travelling from as far away as the University of Würzburg, Germany.

All abstracts accepted for oral and poster presentations are available online, and we encourage delegates to submit their presentations to the ISCB Student Council channel on *F1000Research*- http://f1000research.com/channels/studentcouncil.

### List of keynote speakers

- Professor Veronica van Heyningen, CBE (Honorary Professor, University College London), titled, “Lessons from the genetics of developmental eye disease”.
- Professor Michael Stumpf (Imperial College London) titled, “Recycling, rubbish and waste: proteasome dynamics in health”.
- Dr. Daniel Swan (The Genome Analysis Centre) titled, “How to be a Bioinformatician?”.
- Professor Michael J. E. Sternberg (Imperial College London) titled, “Modelling protein structure and disease-associated missense mutations”.

## Highlights

### Keynotes summary

Prof. van Heyningen delivered a presentation on recent genetic discoveries about developmental eye disorders, an area which she has focused her research on for many years. Her presence, as a pure biologist, was very much appreciated and welcomed by our audience, who were a representative mixture of the interdisciplinarity of bioinformatics. Providing insight into the ‘wet lab’ side of genomics research.

The second keynote speaker, Prof. Michael Stumpf, presented the first comprehensive mathematical model developed in their group which can describe the dynamic proteasomal protein degradation machinery by combining experimental data and computational modelling ^6^. The model suggests that the transport of proteins into the proteasome limits how quickly the proteasome can degrade the proteins, thus revealing differences between the mechanisms of the standard and immuno-proteasomes. Prof. Stumpf highlighted that these findings can help scientists to design drugs for diseases linked to the proteasome machinery that would inhibit the function of immunoproteasomes and not the standard proteasomes.

Dr. Daniel Swan gave a lively and informative talk about his career experiences as his career has progressed. This included practical and wise advice about how to approach the research and development industry, and how to thrive in it. Some of his most memorable pieces of advice were to ‘never stop learning’ and to ‘do what you love’.

Finally, Prof. Michael Sternberg talked about enhanced methods to model protein structures and disease-associated missense mutations. Prof. Sternberg highlighted different computational methods that predict if a missense mutation is associated to a disease and how to identify these using computational tools and resources. The symposium delegates thoroughly enjoyed his keynote and the lively discussion after the talk.

### Student and early-career researchers’ presentations

There were two presentations from post-doctoral research fellows and six presentations from current PhD students. In the first post-doctoral talk, Dr. Pirita Paajanen from The Genome Analysis Centre, UK, discussed her approach to tackling the validation of five ancient DNA samples from Cambridgeshire, UK, and her transition from mathematical thinking to bioinformatics. This presentation was based on the pre-print of a study investigating the impact of Anglo-Saxon migration on the current British population, using a genomics-based approach ^7^. Dr. Paajanen addressed fundamental questions regarding our thinking about proof in bioinformatics compared to mathematics and the good experimental practices that can be applied to the field.

The second post-doctoral talk was delivered by Dr. Carl Barton, Queen Mary University of London, UK. Currently, the effect of epigenetics on aging, in any species, is not well understood. Dr. Barton presented a detailed assessment of epigenetic data provided to him from samples from dogs, and evaluated the statistical methods he used to analyse it.

In the student presentation section, Damjan Temelkovski, University of Westminster, UK, demonstrated his open-source cloud-based tool, Raccoon2, for the simulation of ligand-receptor binding interactions.

Charles Breeze, University College London, UK, presented eFORGE, a tool designed to aid and improve analysis of tissue specific markers in epigenome-wide association studies.

Henry Martell, University of Kent, UK, gave a fascinating talk on the role of assembly in cytogenetics of avian genomes and how this impacts our current understanding of avian evolution.

Matthias Haimel, University of Cambridge, UK, showed us his integrated platform for analysing large sets of variation and phenotypic data, and the potential of this in exploring the genetic cause of rare diseases in patients.

Ananth Surappa-Narayanappa, European Bioinformatics Institute, UK, gave us insight into his finding that protein domains can undergo extreme mutations that create functional domains.

Ricardo Humberto Ramirez Gonzalez, The Genome Analysis Centre/John Innes Centre, UK, informed us of his work on the next-generation sequencing of wheat to identify single nucleotide polymorphisms (SNP) associated with yellow stripe rust resistance. He also described PolyMarker, a tool which can assist with designing primers for SNP assays in polyploid plant species.

## Award winners

All delegates were given the chance to vote for the best oral and poster presentation, with members of the RSG-UK committee awarding a second poster presentation. Henry Martell, University of Kent, received the best oral presentation award for his work titled “Assembling and comparing avian genomes by molecular cytogenetics”.

Sherif Abdelkarim, Aberystwyth University received the best poster presentation award for his titled: “Visualisation and taxonomic identification of metagenomic samples using self organising maps” and the second poster award went to Morena Pappalardo, University of Kent, for his poster titled “Conserved differences in protein structure determine the human pathogenicity of Ebola viruses”.

In addition to a gift card for the best oral and poster presentations, all the award winners received a waived article processing fee from F1000Research.

## Conclusion and future directions

Our second iteration of the RSG-UK student symposium was well received by delegates and sponsors. Positive feedback was received for the organisation of the symposium and quality of presentations and science presented. The science presented in particular was diverse and covered many different themes of computational biology. Furthermore, there was plenty of interest received towards supporting RSG-UK and assisting with future events. Overall, the event continued to maintain the high standards set by the previous year.

Following our successful delivery of two student symposiums, we are now keen to explore the possibility of organising another. Additionally, RSG-UK would like to establish a student representative scheme at universities and institutes in the UK to assist with expanding the RSG-UK member base. RSG-UK is also aiming to arrange smaller career workshops over the next year to support soft skills development. Further details regarding membership and future events will be available on the RSG-UK website - http://rsg-uk.iscbsc.org/.
